# A novel cuproptosis-related prognostic gene signature and validation of differential expression in hepatocellular carcinoma

**DOI:** 10.3389/fphar.2022.1081952

**Published:** 2023-01-10

**Authors:** Yaoting Li, Xuezhen Zeng

**Affiliations:** ^1^ Department of Forensic Science, Guangdong Police College, Guangzhou, Guangdong, China; ^2^ School of Biomedical Sciences, The Chinese University of Hong Kong, Hong Kong, Hong Kong SAR, China; ^3^ Department of Pharmacy, The First Affiliated Hospital of Sun Yat-sen University, Guangzhou, Guangdong, China

**Keywords:** cuproptosis, hepatocellular carcinoma, prognostic model, tumor immunity, drug sensitivity

## Abstract

**Background:** Cuproptosis is a newly discovered form of programmed cell death, which is characterized by accumulation of intra-cellular copper ion leading to the aggregation of lipoproteins and destabilization of Fe-S cluster proteins in mitochondrial metabolism, thereby affecting the prognosis of patients with cancer. However, the role of cuproptosis-related genes (CRGs) in hepatocellular carcinoma (HCC) remains elusive.

**Methods:** Mutation signature, copy number variation and the expression of 10 CRGs were assessed in HCC from TCGA-LIHC dataset. ICGC-LIRI-JP dataset was used as further validation cohort. The least absolute shrinkage and selection operator (LASSO) was used to construct the prognostic model. Kaplan Meier curves, time-ROC curves, nomogram, univariate and multivariate Cox regression were utilized to evaluate the predictive efficacy of CRGs-score. Immune infiltration was analyzed by CIBERSOFT, ssGSEA algorithm, and TIMER database. The expression of prognostic CRGs was validated by qPCR both *in-vitro* and *in-vivo*. Drug sensitivity analysis was performed by pRRophetic.

**Results:** All of the CRGs were differentially expressed in HCC and 5 out of them (CDKN2A, DLAT, GLS, LIPT1, MTF1) correlated with patient survival. These signature genes were selected by LASSO analysis to establish a prognosis model to stratify HCC patients into high and low CRGs-score subgroups. High CRGs-score was associated with a worse prognosis. Subsequently, univariate and multivariate Cox regression verified that CRGs-score was an independent cancer risk factor that correlated with clinical factors including stage and grade. Nomogram integrating the CRGs-score and clinical risk factors performed well to predict patient survival. Immune infiltration analysis further revealed that the expression of immune checkpoint genes was significantly enhanced in high CRGs-score group, especially PD-1 and PD-L1. An independent validation cohort (ICGC) confirmed that CRGs-score as a stable and universally applicable indicator in predicting HCC patient survival. Concordantly, the expression of five confirmed signature genes were also differentially expressed in human HCC cell lines and mouse HCC model. In addition, we also analyzed the sensitivity of 10 clinical targeted therapies between high and low CRGs-score groups.

**Conclusion:** This study elucidated the role of dysregulated CRGs in HCC cohort, with validation with *in-vitro* and *in-vivo* models. The CRGs-score might be applied as a novel prognostic factor in HCC.

## 1 Introduction

Hepatocellular carcinoma (HCC) is the most prevalent primary liver cancer and is the fourth leading cause of cancer-related death worldwide (Kim and Viatour, 2020). HCC is asymptomatic in its early stage, and consequently most HCC patients were diagnosed at advanced stage with limited therapeutic options (Ferrante et al., 2020). Better understanding of HCC may contribute to the development of efficient therapies. Extensive studies have investigated the complex genomic landscape of HCC and demonstrated the correlation between mutated pathways and patient prognosis (Kim and Viatour, 2020; Takeda et al., 2022). Systemic therapies targeting these mutations have been developed, such as Sorafenib, Lenvatinib, bevacizumab, *etc.* However, the prognosis of advanced HCC remains poor (Shah et al., 2017; European Association for the Study of the Liver, 2018; Heimbach et al., 2018). There is an urgent need to explore more novel and efficient targets.

Copper (Cu) is essential trace element that plays pivotal role in enzymatic activity for all organisms (Kim et al., 2008). Genetic variation in copper homeostasis leads to severe disease (Ge et al., 2022). Normally, the concentration of copper in cells are controlled at low levels by active homeostatic mechanisms, which reduces cytotoxicity caused by accumulation of high level of free intracellular copper to cells (Kim et al., 2008; Ge et al., 2022).

Interestingly, accumulating studies showed that the copper levels in both serum and tumor tissue are elevated in a variety of cancers, including lung cancer, breast cancer, cervical cancer, ovarian cancer, prostate cancer, gastric cancer, colorectal cancer, hematological malignancies, *etc* (Gupte and Mumper, 2009; Kaiafa et al., 2012; Zhang and Yang, 2018). In the past recent years, the role of copper in cancers have been extensively explored. It has been reported that copper plays a crucial role in cancer development and progression. As a co-factor for cytochrome C oxidase and lysyl oxidase (LOX) proteins, copper regulates energy production to sustain cancer cell proliferation, invasion and metastasis (Ge et al., 2022). LOX proteins shape the pre-metastatic niches by recruiting myeloid cells to the metastatic sites to form the immunosuppressive and growth-favorable microenvironment for metastasis (Ge et al., 2022). In addition, high levels of copper in cancer cells activate ULK1 and two kinases that regulate autophagy pathway, which enables cancer cells to resist cell death. Copper can also promote angiogenesis in tumor for the transport of nutrients and metabolic wastes (Ge et al., 2022). These studies indicate that copper homeostasis is a potential target for cancer treatment. Therefore, both copper ionophores (e.g. disulfiram, dithiocarbamates, elesclomol, *etc.*) and copper chelators (e.g. trientine, tetrathiomolybdate, *etc.*) have been suggested as cancer therapies (Tisato et al., 2010; Oliveri, 2022). These two types of compounds act in different way. The chelators mainly disrupt the pathways that contribute to cancer progression, while ionophores induce toxic levels of copper ion into the cell that initiate copper-induced cell death. This novel form of cell death is recently reported by Tsvetkov *et al.* in *Science* and termed as “cuproptosis” (Tsvetkov et al., 2022). Similarly, other types of cell death, namely, apoptosis, ferroptosis, necroptosis, and pyroptosis have been discovered and the mechanisms have been well explored. However, mechanisms of cuproptosis remain largely unknown. Tsvetkov *et al.* reported that cuproptosis is regulated by protein lipoylation involved in the tricarboxylic acid (TCA) cycle. Given that lipoylated enzymes are increased in respiring and TCA-cycle active cells, it leads to lipoylated protein aggregation, depletion of Fe-S cluster–containing proteins, and induction of HSP70, which results in acute proteotoxic stress. In this process, ferredoxin 1 (FDX1) is found to be the core mediator of protein lipoylation, and lipoic acid genes are important regulators of cuproptosis (Tsvetkov et al., 2022).

In breast cancer, copper depletion by chelator could reduce metastasis in mouse and human triple negative breast cancer (TNBC) cell lines. Mechanistically, copper chelator decreased oxygen consumption and oxidative phosphorylation, leading to a metabolic switch to glycolysis and reduction of ATP production in TNBC cells (Cui et al., 2021). In addition, multiple studies have identified the key genes involved in cuproptosis in various cancers, which may better predict prognosis of cancer patients and contribute to the development of therapeutic strategies (Bian et al., 2022; Xu et al., 2022).

Given the novel and critical role of cuproptosis in caners, in this study, we aimed to systematically investigate the molecular functions and clinical relevance of cuproptosis-related genes (CRGs) in HCC. We analyzed the data of 368 HCC patients from TCGA database and 240 HCC patients from ICGC database and showed the mutation landscape, expression, functional enrichment analysis of CRGs. Notably, five CRGs were identified and associated with HCC patient survival and prognosis. The prognostic CRGs scoring model based on these genes well predicted patient survival, which was also validated by external HCC patient cohort. The expression of these genes was validated *in vitro* and *in vivo* HCC models. In addition, immune cell landscape and drug sensitivity were also analyzed. Collectively, our study comprehensively analyzed the role of CRGs in different aspects of HCC and highlights the importance of CRGs in HCC development, which provide the knowledge for the therapeutic application of cuproptosis related signature in HCC.

## 2 Methods

### 2.1 Datasets and preprocessing

The RNA-sequencing data (FPKM value) of 374 HCC patients and the corresponding clinical information were downloaded from The Cancer Genome Atlas (TCGA) database (https://portal.gdc.cancer.gov/, accessed in July 2022). The mRNA expression data and clinical information of ICGC-LIRI-JP dataset were downloaded from International Cancer Genomics Consortium (ICGC) website. Patients without survival information were excluded for further analysis. A total of 368 HCC patients and 50 adjunct non-tumor samples in TCGA and 240 HCC patients in ICGC were involved in the present study. The clinical information of HCC samples was listed in Supplementary Table S1. For data normalization, the values of FPKM data in two cohorts were transformed into transcripts per million kilobase (TPM) values. Furthermore, somatic mutations data and copy number variation (CNV) data were also obtained from TCGA database. This study was based on public databases, the approval of the local ethics committee was not required. Data analysis was performed with the R (version 4.2.1), R-studio and Bioconductor packages.

### 2.2 Cell culture

Human normal liver cells (LO2) and Hepatocellular Carcinoma cells (Huh7 and MHCC97H) were purchased from Shanghai Cell Bank of Chinese Academy of Sciences (China), which were maintained in DMEM plus 10% FBS (Gibco, United states) and 1% penicillin-streptomycin. All these cell lines were incubated at 37 °C under the condition of 5% CO2.

### 2.3 Hydrodynamic injection of AKT/NRAS plasmid to induce mouse spontaneous HCC

Male C57BL/6 mice of 4-week-old were separated into control and HCC group. For HCC group, 20 μg pT3-EF1a-HA-myr-AKT (Addgene), 20 μg pT/Caggs-NRASV12 (Addgene) along with 2.85 μg SB transposase plasmid (Addgene) were diluted in 2.0 mL saline, and then injected into mice by tail vein injection. For control group, equal volume of saline was injected into mice by tail vein injection. All mice were sacrificed to collect liver and tumor tissue 4–6 weeks later.

### 2.4 Identification of cuproptosis-related gene

A total of 10 Cuproptosis-Related Genes (CRGs) were obtained from prior papers (Tsvetkov et al., 2022) and listed in Supplementary Table S2. The differential expression of CRGs between HCC and normal liver tissues were identified using the “limma” package (Ritchie et al., 2015).

### 2.5 Mutation analysis of CRGs

The mutation frequency and waterfall plot of 10 CRGs in HCC patients were generated by the “maftools” package (Mayakonda et al., 2018). The location of 10 CRGs on 23 chromosomes was drawn by the “circlize” package (Gu et al., 2014). The CNV values were set by .3 as a threshold. The Cleveland dot plot was visualized the frequency of CNV by the “ggpubr” package.

### 2.6 Functional enrichment analysis

Gene Ontology (GO) of cuproptosis-related genes was analyzed *via* “clusterProfiler” package (Yu et al., 2012). Similarly, we performed Kyoto Encyclopedia of Genes and Genomes (KEGG) analysis by the same package. We applied the Benjamini−Hochberg method for the multiple correction, and terms with adjusted *p* < .05 were significantly enriched. The results of GO and KEGG were shown in Supplementary Tables S3,S4.

### 2.7 Univariate and multivariate cox regression analysis

We performed the univariate and multivariate Cox regression analysis for overall survival (OS) in HCC clinical data. *p*-values and hazard ratio (HR) with 95% confidence interval (CI) were generated by Cox proportional hazard regression. CRGs with a significant prognostic value were selected for further analysis. The forest plot of each variables was generated through the “forestplot” R package (Dettori et al., 2021).

### 2.8 The establishment of a CRGs scoring model and prognostic analysis

We established a predictive scoring model using LASSO-Cox analysis. CRGs-score = (coefficient A) * gene A+ (coefficient B) * gene B+ …… + (coefficient N) * gene N. where coefficient N, gene N represented the coefficient index, and the gene expression level, respectively. Subsequently, we divided patients into the high-risk score and low-risk score subgroups according to the median value of CRGs-score, and the overall survival (OS), disease-free interval (DFI), disease-specific survival (DSS), and progression-free interval (PFI) were compared between the two subgroups *via* Kaplan-Meier analysis. The predictive accuracy of this model was evaluated by performing time-dependent receiver operating characteristic (ROC) analysis (Kamarudin et al., 2017).

### 2.9 Nomogram construction

Independent prognostic variables were integrated to construct the nomogram in predicting 1-, 3-, and 5-year overall survival time with stepwise Cox regression analysis. The calibration curves of the CRGs-score and other clinical indicators were estimated using 500 bootstrapping to determine bias-corrected estimates of predicted *versus* actual values. The curves were plotted to compare nomogram-predicted and observed 1-, 3-, and 5-year survival time.

### 2.10 Tumor microenvironment cell infiltration analysis

We performed the CIBERSORT algorithm to quantify the proportion of immune cells between CRGs-score subgroups. We calculated the normalized gene expression data by LM22 signature and 500 permutations. Then we performed pathway enrichment analysis by the “GSVA” package. We downloaded immune associated signatures to carry out GSVA analysis. Moreover, we examined seven important immune checkpoint molecules including CTLA4, HAVCR2, LAG3, PD-L1, PD-1, PDCD1LG2, TIGIT between two subtypes. The TIMER database (cistrome.shinyapps.io/timer) (Li et al., 2017) was used to investigate the correlation between the expression of CRGs and the abundance of six immune cells (B Cells, CD8^+^ T-cell, CD4^+^ T-cell, macrophages, neutrophils, and dendritic cells).

### 2.11 RNA extraction and quantitative real-time PCR

Total RNA was extracted by Trizol reagent (Invitrogen, United states) according to the manufacturer’s protocol. Then, the RNA was reverse transcribed using PrimeScript RT reagent Kit with gDNA Eraser (Takara, Japan). The cDNAs were subjected to SYBR Green dye qPCR analysis. The primers used in real-time qPCR assays were listed in Supplementary Table S5.

### 2.12 Drug sensitivity analysis

The “pRRophetic” package was used to analyze the drug sensitive prediction based on the Genomics of Drug Sensitivity in Cancer (GDSC) database. The half-maximal inhibitory concentrations (IC50) of the samples were calculated by ridge regression algorithm (Geeleher et al., 2014) and the difference of anticancer drug sensitivity were compared between the high CRGs and low CRGs-score group.

### 2.13 Statistical analysis and cut-off value

The log-rank test and “ggsurvplot” package were utilized to perform the survival analysis. The cut-off value was 2.309 which was the median value of CRGs-score. The risk factors and drug sensitivity analysis between the two groups were performed using the Wilcox test. QPCR data of validation experiments was calculated in GraphPad Prism eight software. The independent Student’s t-test was applied to compare data between two subgroups. All *p* < .05 represents statistical significance.

## 3 Result

### 3.1 Overview of genetic variation of cuproptosis-related genes in HCC

**FIGURE 1:**
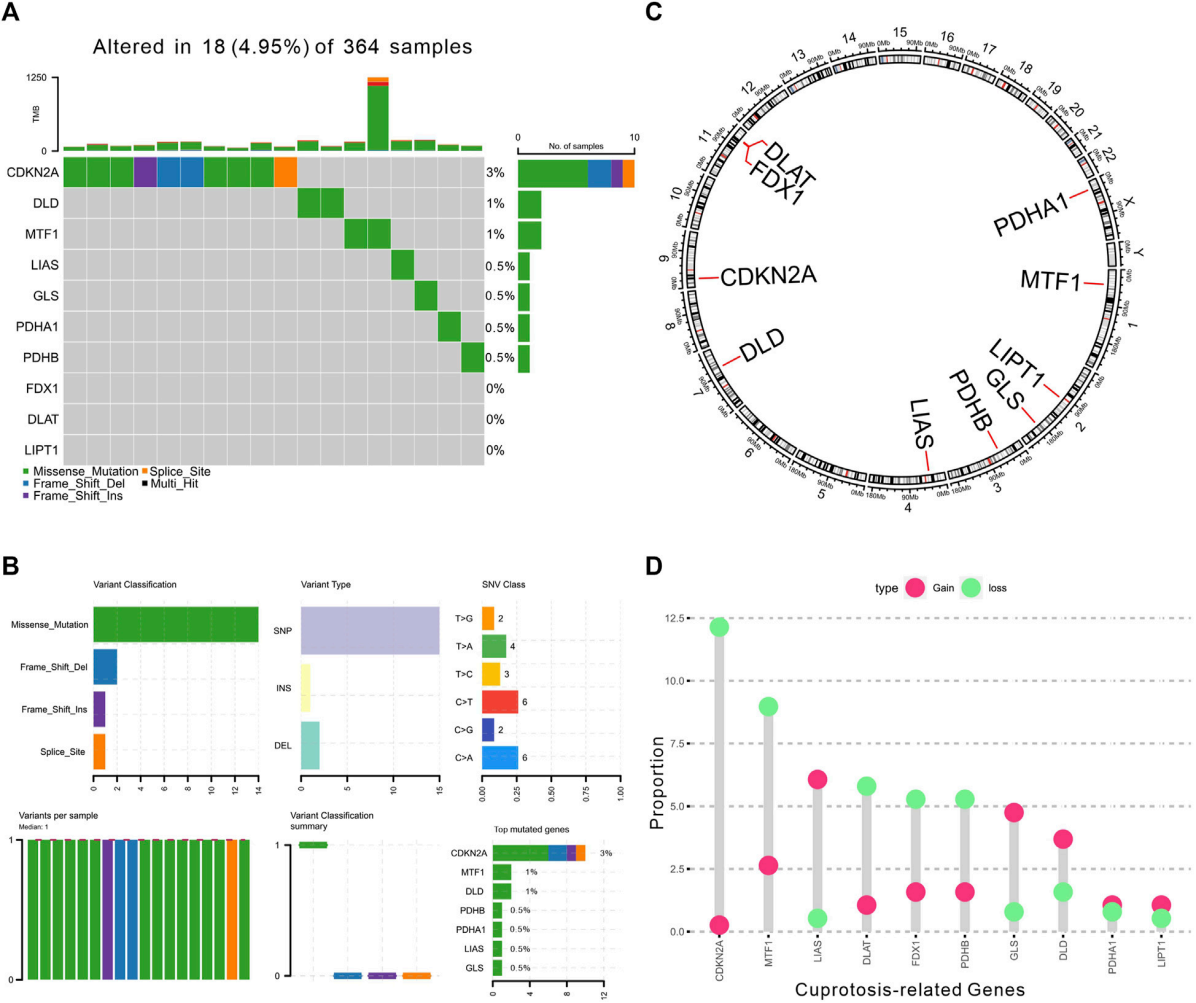
Landscape of genetic alterations of CRGs in HCC. **(A)** The landscape of mutation profiles of 364 HCC patients from TCGA-LIHC cohort. The upper barplot represented the mutation burden. The right barplot showed mutation frequency individually. **(B)** The mutation summary plot of CRGs. The barplot displayed the variant classification, variant types, SNV class, and top mutated CRGs. **(C)** The location of CNV alteration of 10 CRGs on 23 chromosomes. **(D)** The CNV frequency of CRGs in TCGA cohort. The height of the column showed the proportions of gain or loss variations.

The prevalence of Cuproptosis-Related Genes (CRGs) in HCC was determined focusing on somatic mutations and copy number variations (CNVs). At the genetic level, mutation of CRGs was observed in 18 of 364 (4.95%) HCC samples (Figure 1A). Among different classification of mutations, we found that missense mutation was most frequently observed. And single nucleotide polymorphism (SNP) was the most prevalent variant type. C > T and C > A were both ranked as the top single nucleotide variants (SNV) class. Of these, we also found that CDKN2A (3%), MTF1 (1%) and DLD (1%) showed higher mutation frequencies than other genes (Figure 1B). In addition, the location of CNV alterations of these 10 CRGs on chromosomes were shown in Figure 1C. However, the CNV alterations were not universally prevalent among these CRGs. Specifically, LIAS, GLS, and DLD showed distinctive copy number amplification, while CDKN2A, MTF1, DLAT, FDX1, and PDHB exhibited significant CNV deletion (frequency >3%, Figure 1D), indicating that changes in CNVs of CRGs may be important factors leading to abnormal gene expression.

### 3.2 Functional enrichment analysis of CRGs

**FIGURE 2:**
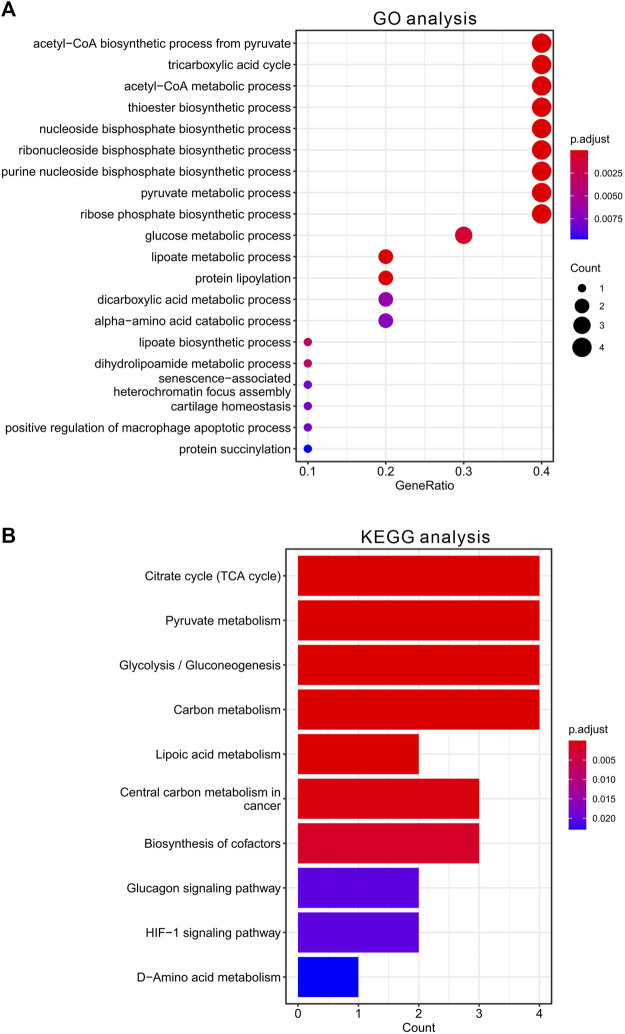
Functional enrichment analysis of CRGs in HCC. **(A)** The top 20 enriched items of CRGs in gene ontology analysis in biological process. The size of circles represented the number of genes enriched. **(B)** The top 10 enriched pathways of CRGs in KEGG database.

To further clarify the function of CRGs, we further performed enrichment analysis using GO and KEGG database. The 10 CRGs were mainly involved in acetyl-CoA biosynthetic and metabolic process from pyruvate, TCA cycle, thioester biosynthetic process, nucleoside and ribonucleoside bisphosphate biosynthetic process, pyruvate metabolic process, glucose metabolic process, lipoate metabolic process, protein lipoylation, dicarboxylic acid metabolic process, alpha-amino acid catabolic process, dihydrolipoamide metabolic process, senescence-associated heterochromatin focus assembly, cartilage homeostasis, positive regulation of macrophage apoptotic process, protein succinylation in GO analysis (Figure 2A). KEGG pathway analysis also suggested that these CRGs were mainly involved in TCA cycle, glycolysis, pyruvate and carbon metabolism, lipoic acid metabolism, central carbon metabolism in cancer, biosynthesis of cofactors, glucagon signaling pathway, HIF-1 signaling pathway, D-Amino acid metabolism (Figure 2B, data was shown in Supplementary Tables S3,S4 **)**.

### 3.3 Determination of differentially expressed and prognostic CRGs in HCC

**FIGURE 3:**
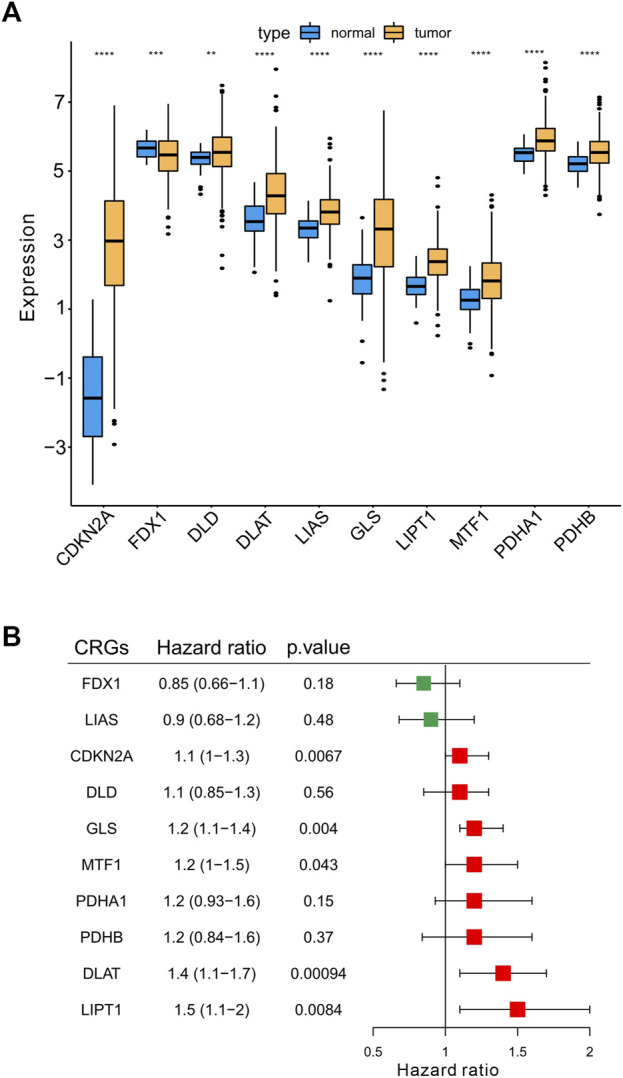
Determination the differentially expressed CRGs and prognostic signatures in HCC. **(A)** The expression of 10 CRGs in HCC (*n* = 368) and normal liver tissues (*n* = 50) in TCGA-LIHC cohort. Tumor was shown in orange, while normal liver was shown in blue (*t*-test, *p < .05; **p < .01; ***p < .001; ****p < .0001; ns, not statistically significant). **(B)** Univariate Cox regression analysis of CRGs. The right boxplots represented the Hazard ratio with 95% confidence interval, the *p*-value was calculated by univariate cox regression.

Next, we explored the expression of the 10 CRGs in 368 HCC samples (6 samples without clinical information were excluded for this analysis) and normal liver tissue (50 samples) using the TCGA-LIHC dataset. We showed that these 10 CRGs were differentially expressed (either upregulated or downregulated) in HCC compared to normal liver (Figure 3A). Specifically, the expression of CDKN2A, DLD, DLAT, LIAS, GLS, LIPT1, MTF1, PDHA1, and PDHB was upregulated, while the expression of FDX1 was significantly downregulated in HCC. Moreover, we analyzed the prognostic values of CRGs by univariate Cox regression analysis. A total of five genes (CDKN2A, GLS, MTF1, DLAT, LIPT1) with a significant *p*-value were identified (*p* < .05). These five genes were associated with increased risk of HCC with HRs >1 and recognized as prognostic CRGs (Figure 3B).

### 3.4 Development of the prognostic CRGs scoring model

**FIGURE 4:**
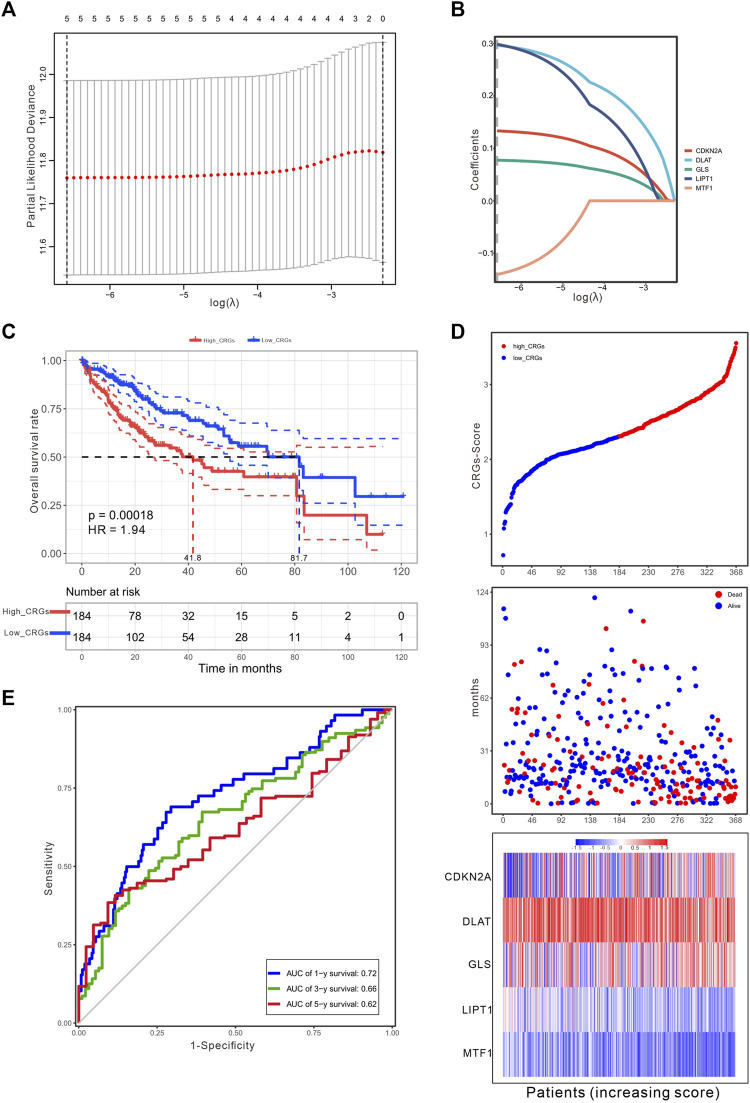
Construction of a prognostic CRGs model. **(A)** The LASSO-COX model screened out prognostic CRGs and carried out 10-fold cross-validation. The λ value was confirmed as .0014 where the optimal lambda resulted in five non-zero coefficients. **(B)** LASSO coefficient profiles of the five CRGs. **(C)** The survival curves for the different CRGs-score subgroups with the cut-off value 2.309 among 368 HCC patients (Log-rank test, *p* = .00018, HR = 1.94). The mean OS for the high and low CRGs-score group were 41.8 and 81.7 months, respectively. **(D)** The distribution of CRGs-score, survival status, and the expression of five prognostic CRGs in HCC. **(E)** The time-dependent receiver operating characteristic (ROC) analysis of CRGs-score. The AUC values were .72, .66, .62 at 1 year, 3 years, and 5 years, respectively.

To establish a prognostic gene scoring model, LASSO-Cox regression analysis was performed based on the above five prognostic CRGs, and finally all these genes were selected according to the minimum criteria (Figures 4A,B). CRGs-score was calculated by CRGs and the corresponding coefficients (Table 1). Next, we divided the HCC patients into low and high CRGs-score subgroups with the median value of 2.309. The Kaplan-Meier (KM) curve revealed that HCC patients with high CRGs-score had a worse overall survival than those with low CRGs-score (median time = 41.8 vs. 81.7 months, *p* = .00018, HR = 1.94, Figure 4C). With the CRGs-score increasing, the survival status of patients, and genes expression pattern of these five genes are presented in Figure 4D. The results showed that increased CRGs-score was accompanied by decreased survival and increased risk of death. The expression of CDKN2A, DLAT, and GLS was higher, while MTF1 was lower in high CRGs-score compared to low CRGs-score (Figure 4D). Furthermore, we applied a weighted CRGs-score incorporating all related genes to estimate 1-, 3- and 5-year overall survival. The prediction accuracy evaluated by AUCs was .72, .66, and .62 in the 1-year, 3-year and 5-year ROC curves, respectively (Figure 4E).

**TABLE 1:** Cuproptosis-related signature genes and coefficients.

Gene	Coefficient
CDKN2A	.1335
DLAT	.2969
GLS	.0775
LIPT1	.2988
MTF1	−.1407

### 3.5 The CRGs-Score indicated prognosis and clinical features

**FIGURE 5:**
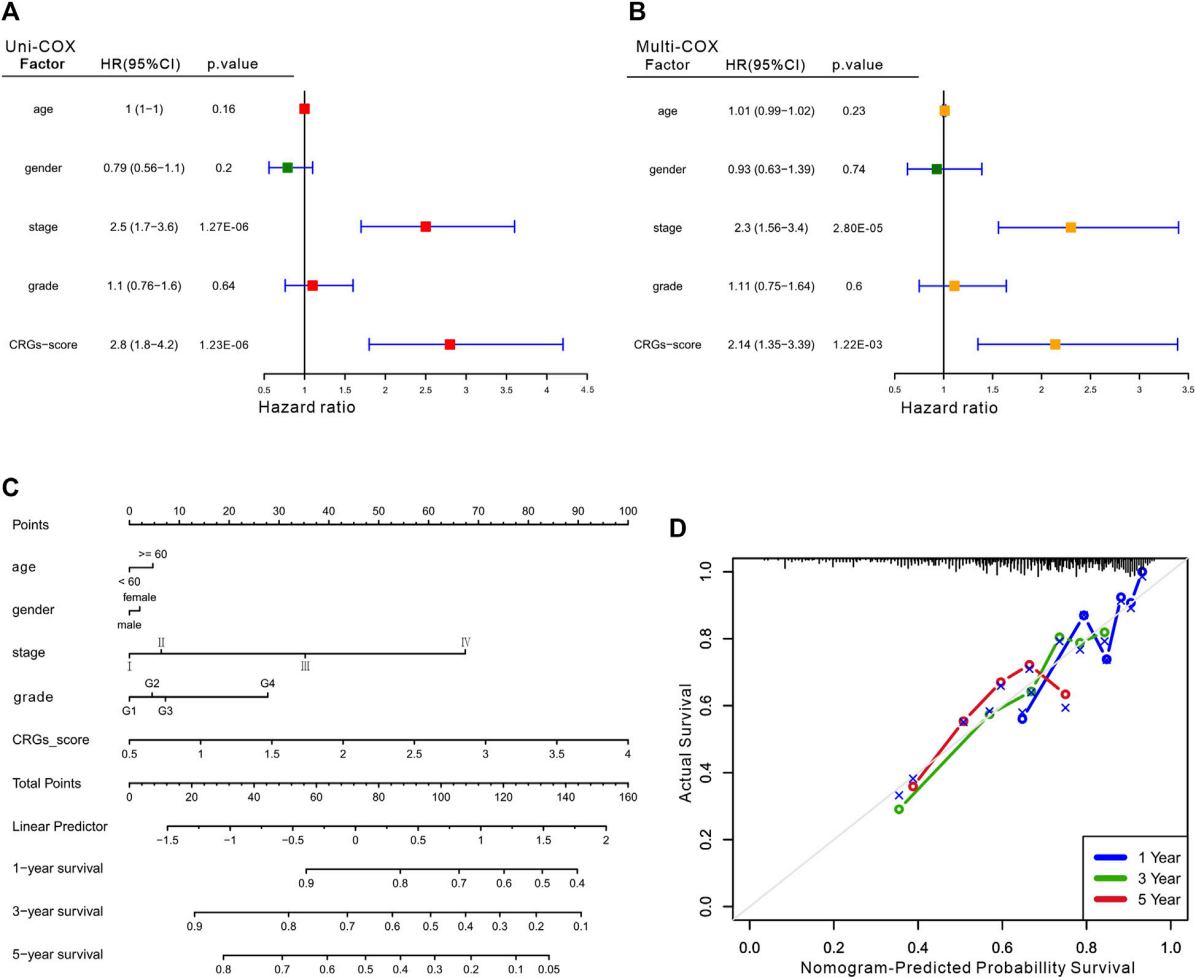
The clinical features of the CRGs-score model. The forest plot for univariate Cox **(A)** and multivariate Cox regression **(B)** considering clinical indicators and CRGs-score in HCC cohort. **(C)** Nomogram incorporating age, gender, stage, grade and CRGs-score was a predictor of 1-, 3-, and 5-year overall survival probabilities in HCC patients. **(D)** Calibration curves of 1-, 3-, and 5-year of survival outcomes.

**FIGURE 6:**
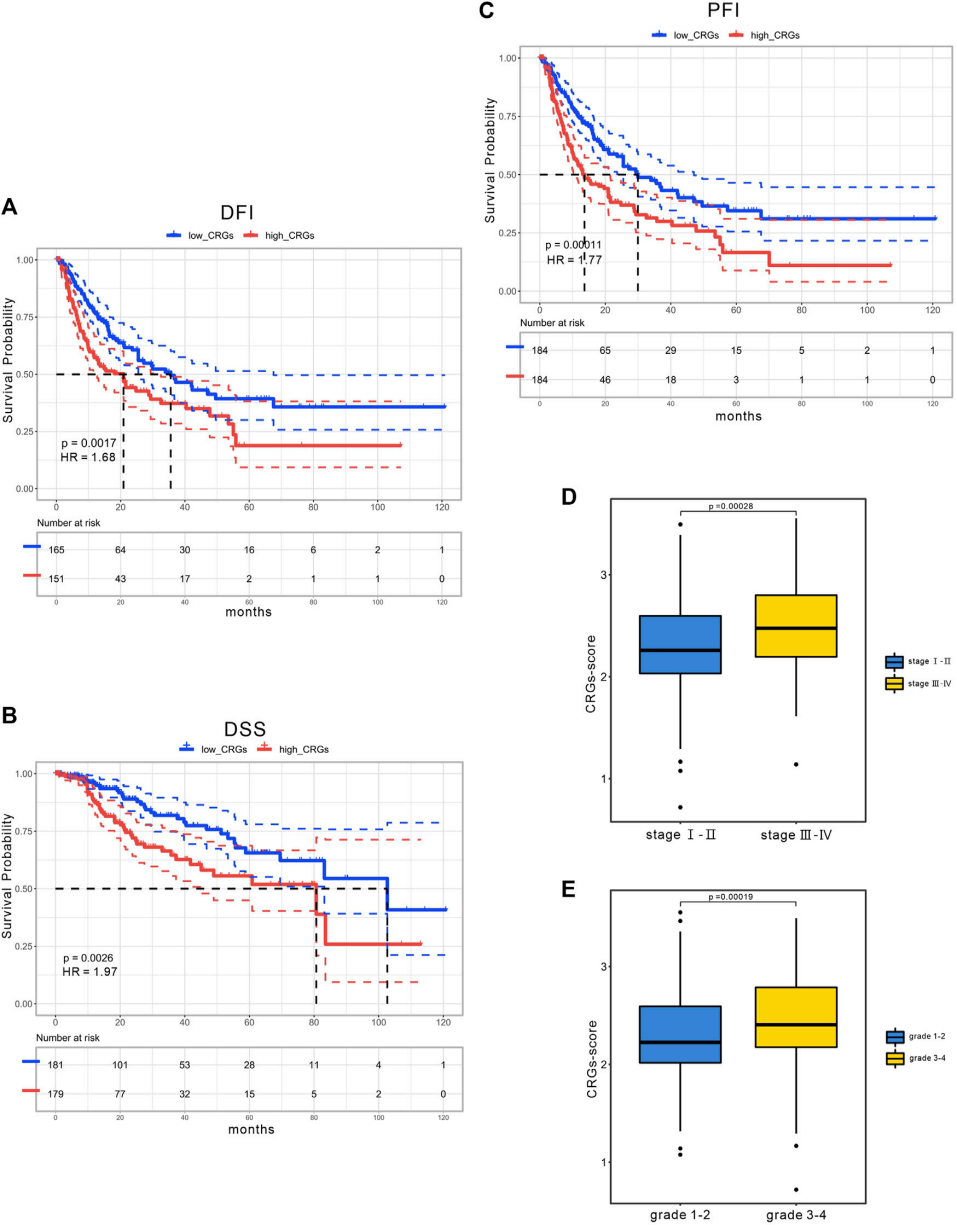
The clinical application of CRGs-score. The KM curves of **(A)** DFI **(B)** DSS **(C)** PFI time between low and high CRGs-score subgroups in the TCGA cohort. **(D)** CRGs-score between the early stage and the advanced stage subgroups. **(E)** CRGs-score between the low-grade and the high-grade subgroups.

Considering the importance of the CRGs-score in evaluating the prognosis of HCC patients, we next investigated its applied value in clinical diagnosis. Univariate and multivariate COX analysis revealed that stage and CRGs-score were independent factors affecting HCC patient’s prognosis (Figures 5A,B). We also constructed a nomogram featuring age, gender, stage, grade and CRGs-score to predict the survival rate of HCC patients at 1, 3, and 5 years (Figure 5C). Intriguingly, the nomogram exhibited good predictive value with a C-index of .67. The calibration of the 1-year, and 3-year overall survival rates were relatively well compared with an ideal model in the entire cohort (Figure 5D). In the KM curves, the disease-free interval (DFI), disease-specific survival (DSS) and progression-free interval (PFI) in the high CRGs-score group were all significantly lower than those in the low CRGs-score group, with the HRs of 1.68, 1.97, and 1.77 respectively (Figures 6A–C). Moreover, patients with advanced stage or higher grade had a higher CRGs-score significantly (Figures 6D,E). These results indicate that the CRGs-score is an excellent predictive prognostic trait.

### 3.6 Association between the CRGs prognostic signatures and immune microenvironment

**FIGURE 7:**
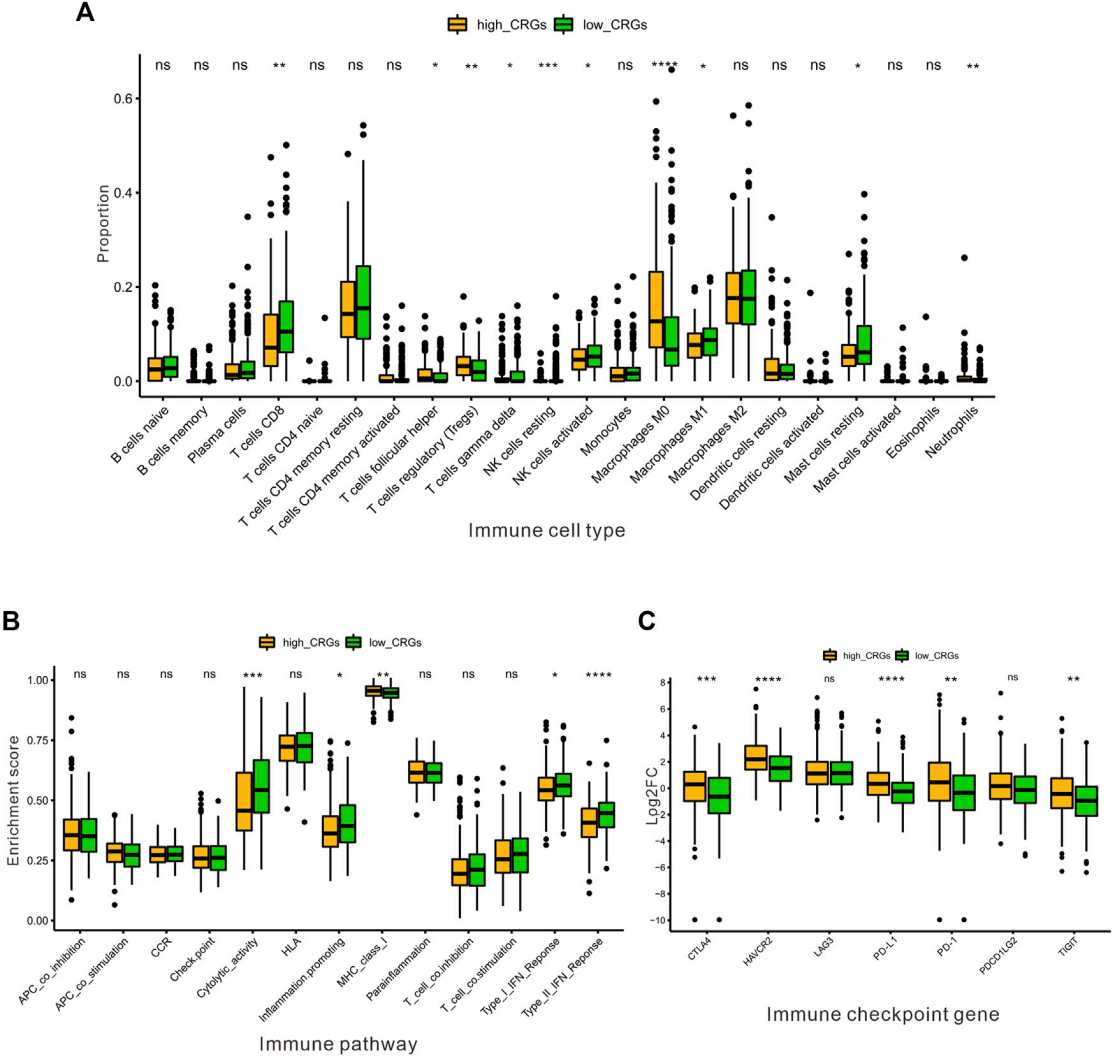
The association between the CRGs and immune microenvironment in HCC. **(A)** The proportion of immune infiltrating cells between the low and the high CRGs-score subgroups by CIBERSOFT analysis. **(B)** The enrichment score of thirteen immune pathways between the low and the high CRGs-score groups. **(C)** The boxplots were utilized for visualizing the expression of seven immune checkpoint genes in the low and the high CRGs-score subgroups.

**FIGURE 8:**
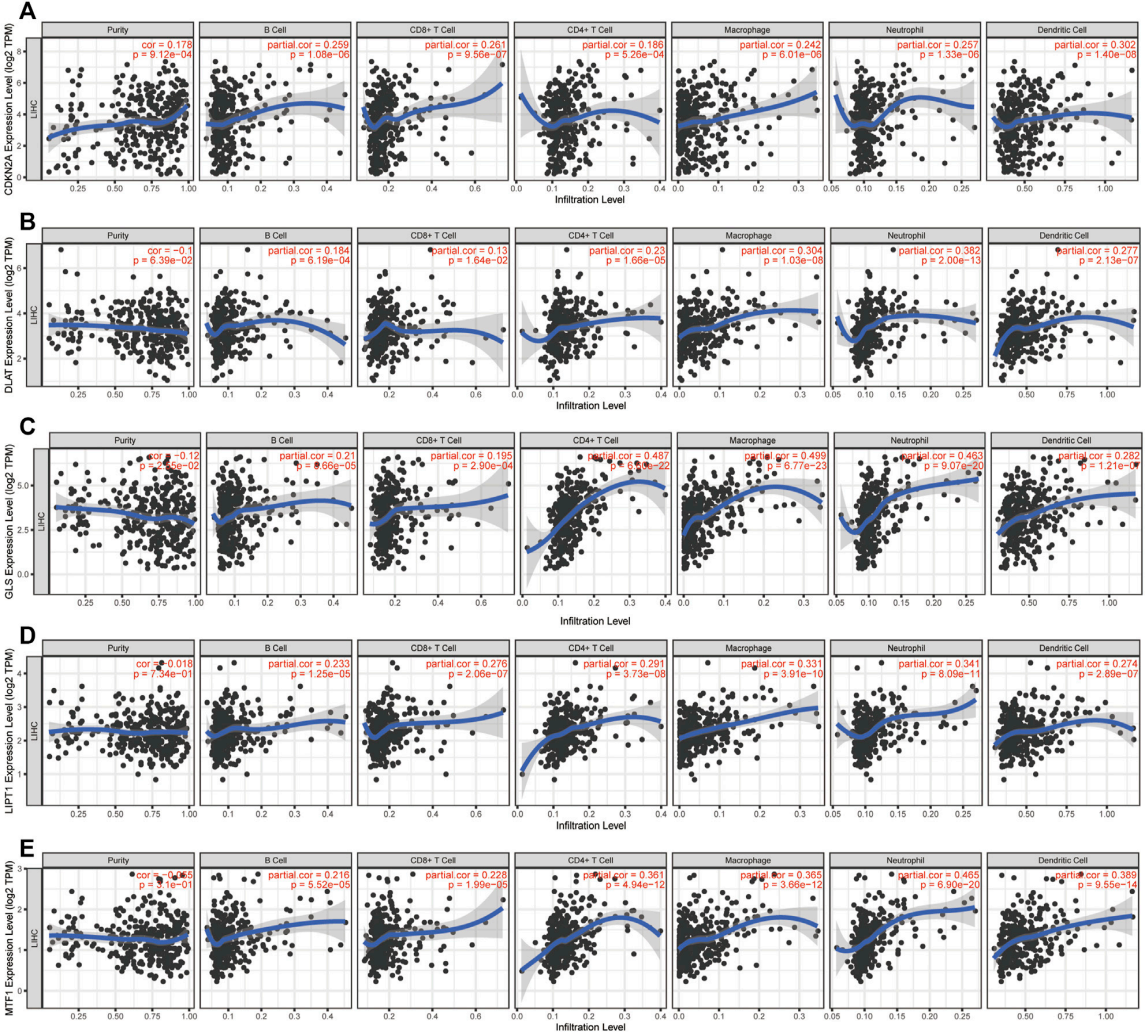
Immune infiltration analysis of CRGs signatures. Correlation between **(A)** CDKN2A **(B)** DLAT **(C)** GLS **(D)** LIPT1, and **(E)** MTF1 expression and immune infiltration in HCC from the TIMER database.

Next, we further assessed whether the CRGs signature affected tumor immunity. Immune infiltration analysis was performed using CIBERSOFT algorithm. We found that the proportion of CD8^+^ T-cell, gamma delta T-cell, nature Killer cells, M1 macrophage and resting Mast cells were relatively higher in low CRGs-score group, while the proportion of follicular helper T-cell, regulatory T-cell, M0 macrophage, and neutrophils cells were lower in low CRGs-score group compared to high CRGs-score group (*p* < .05, Figure 7A). Additionally, we also analyzed thirteen immune related pathways between high and low CRGs-score subgroups by ssGSEA method. The high CRGs-score group exhibited downregulation of cytolytic activity, inflammation promoting, type Ⅰ and type Ⅱ IFN response and upregulation of MHC class Ⅰ pathway (*p* < .05, Figure 7B). We next compared the expression of seven immune checkpoint genes between two groups and found that the expression of immune checkpoint molecules including CTLA4, HAVCR2 (also known as TIM-3), LAG3, PD-L1, PD-1, PDCD1LG2 (PD-L2) and TIGIT. Interestingly, we found that CTLA4, HAVCR2, PD-L1, PD-1 and TIGIT were significantly higher in the high CRGs-score group, indicating that HCC in cuproptosis status might be sensitive to immune checkpoint blockade therapies (*p* < .05, Figure 7C). Moreover, we performed a correlation analysis between CDKN2A, DLAT, GLS, LIPT1, MTF1 and different immune cell types. Notably, the expression level of CDKN2A, DLAT, GLS, LIPT1, and MTF1 were positively associated with the immune infiltration level of B-cell, CD8^+^ T-cell, CD4^+^ T-cell, Macrophage, Neutrophil, and Dendritic Cell (all *p* < .05, Figures 8A–E).

### 3.7 Validation of the prognostic signature by independent cohort

**FIGURE 9:**
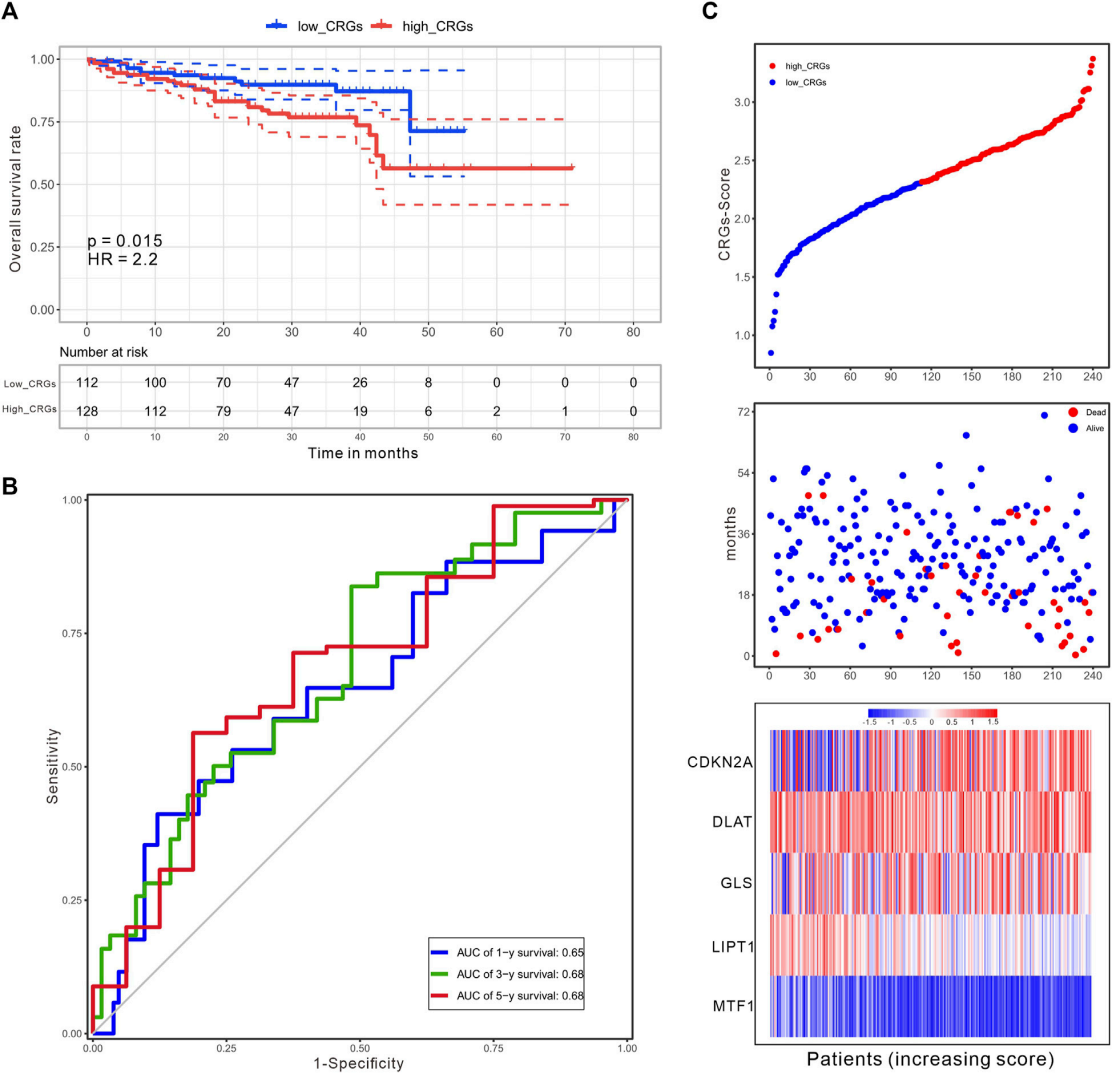
An independent validation of the CRGs-score with the ICGC cohort. **(A)** The KM curves for overall survival of 240 HCC patients between low and high CRGs-score subgroups (Log-rank test, *p* = .015, HR = 2.2). **(B)** The time-dependent ROC analysis of CRGs-score. The AUC values were .65, .68, .68 at 1 year, 3 years, and 5 years, respectively. **(C)** The increasing of CRGs-score, survival status, and heatmap of five prognostic CRGs in the ICGC cohort.

The CRG-score in the ICGC validation cohort (*n* = 240) was calculated with the weighted formula derived from the training cohort, and the HCC patients were also divided into low and high CRGs-score subgroups with the cut-off value (2.309) consistent with the training cohort. The OS of patients with high CRGs-score was obviously decreased (*p* = .015, Figure 9A). The AUC values of OS at 1, 3, and 5 years predicted by the CRGs-score were .65, .68, and .68 respectively (Figure 9B). Consistently, in both cohorts, high CRGs-score correlated with poor survival. The expression of CDKN2A, DLAT, and GLS was increased, while expression of MTF1 was decreased accompanied with raised CRGs-score (Figure 9C).

### 3.8 Validation of the expression of CRGs signatures in HCC *in-vitro* and *in-vivo*

**FIGURE 10:**
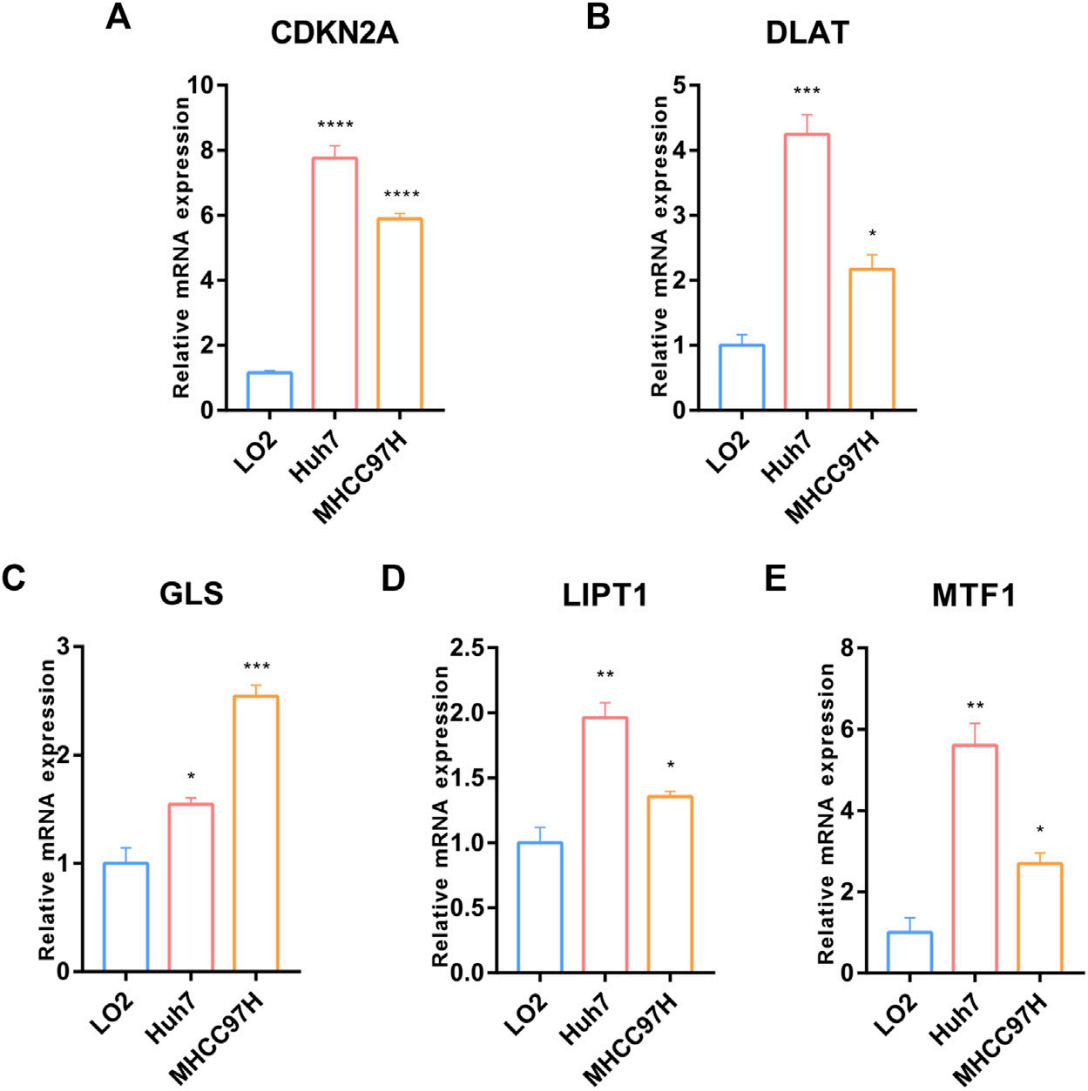
Validation of the expression of the five prognostic CRGs *in-vitro* HCC cell lines model. **(A–E)** RT-qPCR was performed to detect the expression of CDKN2A, DLAT, GLS, LIPT1 and MTF1 in normal liver cell (LO2) and HCC cells (Huh7 and MHCC97H) (Data are presented as mean ± SEM, *t*-test, *p < .05; **p < .01; ***p < .001; ****p < .0001; ns: not statistically significant).

**FIGURE 11:**
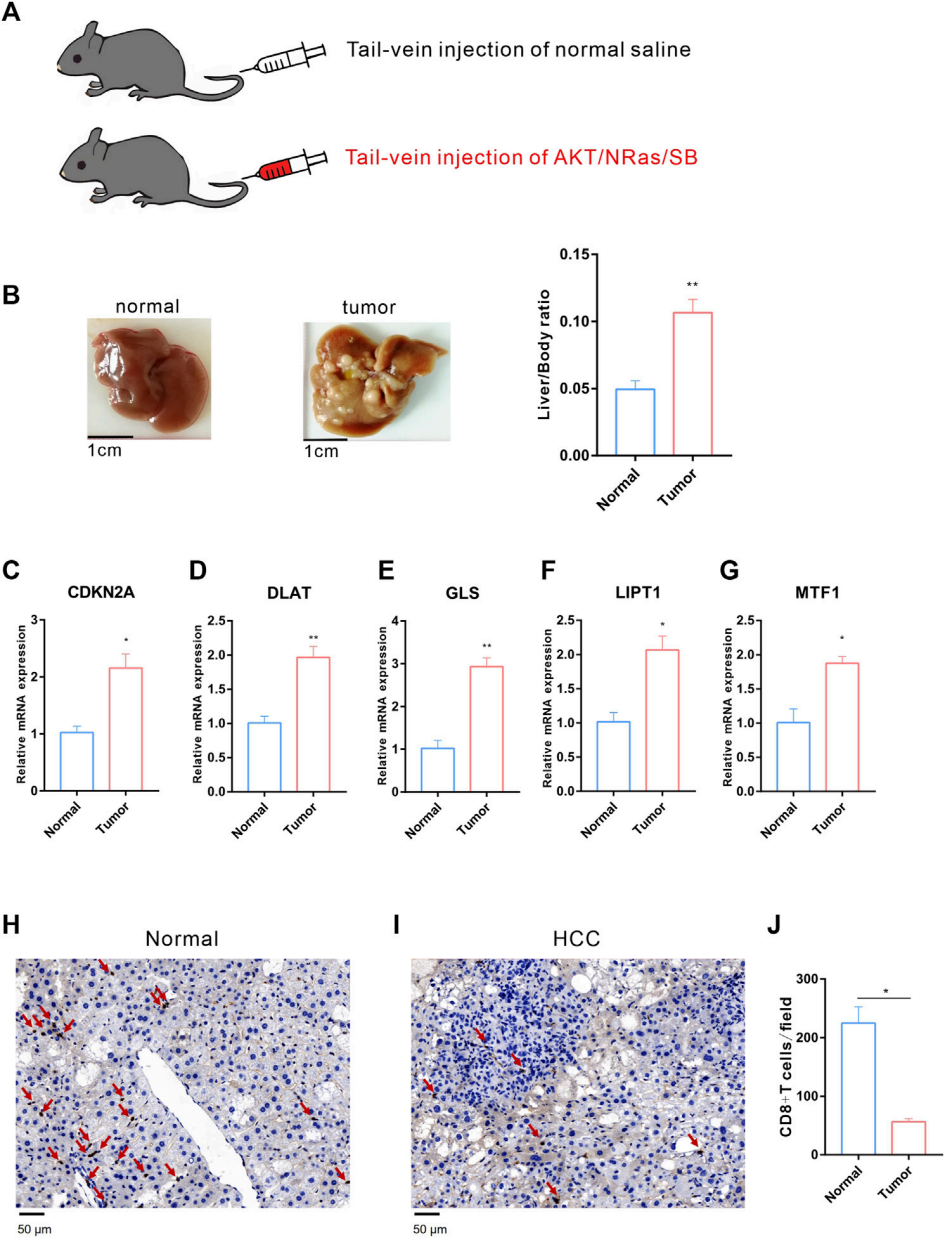
Validation of the expression of the five prognostic CRGs *in vivo* HCC mouse model. **(A)** HCC mouse model diagram: C57BL/6 J male mice were hydrodynamic injected with normal saline or AKT/NRas/SB plasmids, respectively. **(B)** Representative mouse liver tissues of normal (left) and HCC (middle) and liver/body ratio between two groups (right). **(C–G)** The mRNA expression of CDKN2A, DLAT, GLS, LIPT1 and MTF1 in normal and HCC liver. **(H–I)** Immunohistochemistry (IHC) staining of CD8 between normal **(H)** and HCC **(I)** liver tissues. **(J)** The statistical analysis of CD8^+^ T-cell in filed between normal and HCC group.

We further validated the expression of the five prognostic CRGs (CDKN2A, DLAT, GLS, LIPT1, and MTF1, which constructed the CRGs-score model) in HCC cell lines and mouse HCC model. We analyzed the mRNA level of these five genes in the normal liver cell (LO2) and two HCC cells (Huh7, MHCC97H) by RT-qPCR. As shown in Figure 10, the transcription of CDKN2A, DLAT, GLS, LIPT1, and MTF1 were significantly enhanced in Huh7 and MHCC97H cells compared to LO2 cells (Figures 10A–E). We also established spontaneous HCC mouse model by hydrodynamic tail vein delivery of the NRASV12 and myr-AKT in C57BL/6 mice (Figure 11A). Mice injected with NRASV12 and myr-AKT plasmids successfully developed HCC tumors and exhibited higher liver/body ratio than normal group (Figure 11B). Additionally, increased mRNA level of CDKN2A, DLAT, GLS, LIPT1, and MTF1 in tumor were observed compared to normal group (Figures 11C–G). Furthermore, to investigate tumor immunity, we collected the HCC and normal tissues and detected CD8 expression by Immunohistochemistry (IHC) staining. Notably, CD8^+^T-cell were significantly decreased in HCC compared to normal group (Figures 11H–J).

### 3.9 Drug sensitivity analysis between the curpoptosis-related subtypes in HCC

**FIGURE 12:**
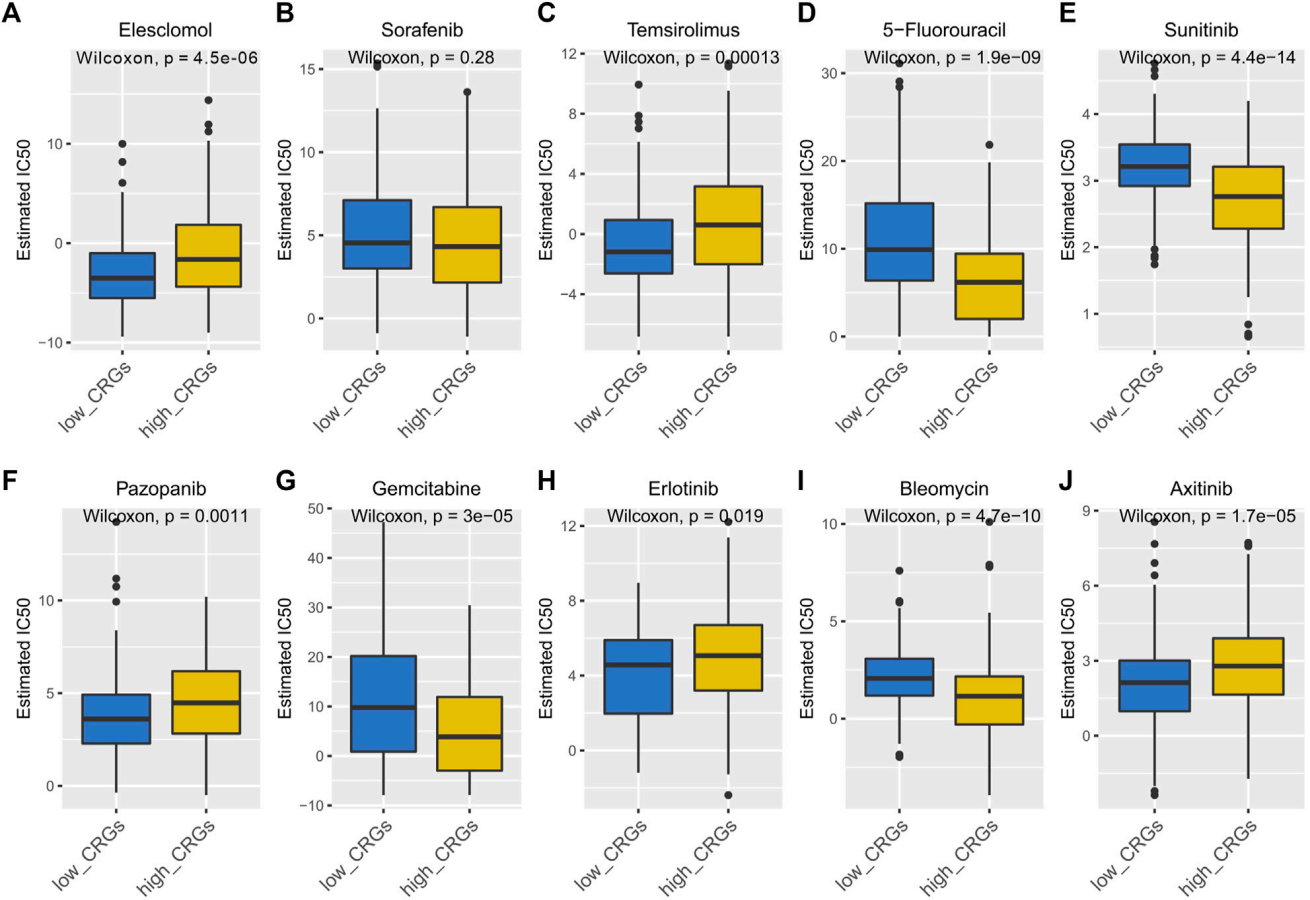
The IC50 of 10 commonly used chemotherapeutic drugs and targeted drugs in the low and high CRGs-score subtypes of HCC. **(A–J)** The IC50 of Elesclomol, Sorafenib, Temsirolimus, 5-Fluorouracil, Sunitinib, Pazopanib, Gemcitabine, Erlotinib, Bleomycin, Axitinib between low and high CRGs-score subgroups.

Elesclomol is an anticancer drug that targets mitochondrial metabolism and plays an important role in transportation copper ions that has been shown to induce cuproptosis. Therefore, we tested the sensitivity of Elesclomol between the low and high CRGs-score subtypes in HCC patients. The elesclomol IC50 of low CRGs-score was significantly lower than high CRGs-score subtype, indicating increased sensitivity (*p* = 4.5e-06, Figure 12A). To clarify the roles of CRGs on sensitivity of chemotherapeutics and molecular targeted drugs, we also revealed the sensitivity for nine commonly used drugs in HCC between low and high CRGs-score subtypes. The results showed that the IC50 of 5-Fluorouracil, Sunitinib, Gemcitabine, and Bleomycin was lower in high CRGs-score subtype. However, the IC50 of Temsirolimus, Pazopanib, Erlotinib, and Axitinib was higher, compared to low CRGs-score subtype in HCC (Figures 12B–J).

## 4 Discussion

Characterized by biological invasiveness and potential metastasis, HCC is still one of the most prevalent cancers leading to cancer-related death. Moreover, the incidence and mortality of HCC are still arising. Currently, the diagnosis and prognosis of HCC patients depend on traditional indicators, such as Cancer of the Liver Italian Program (CLIP) score, Tumor Node Metastasis (TNM) stage, Barcelona Clinic Liver Cancer (BCLC) stage. A more personalized and better staging system may contribute to the development of efficient diagnostic and prognostic biomarkers, precision therapy, prediction of patient prognosis and improved clinical outcome.

A recent study found that the unbalance of intracellular copper ion accumulation triggered aggregation of mitochondrial lipoproteins, leading to a unique type of cell death called cuproptosis (Tsvetkov et al., 2022). Different from other forms of cell death, such as autophagy, necroptosis, and pyroptosis, cuproptosis targeted lipoylated TCA cycle proteins without triggering the caspase 3 activity. Copper acts as a catalytic cofactor for essential enzymes involved in energy conversion, oxygen transport, and regulation of oxidative metabolism in cell activities (Kim et al., 2008). Additionally, Copper also promotes angiogenesis in tumor for the transport of nutrients and metabolic wastes. Imbalance of copper metabolism seriously affects normal metabolism of the liver, which leads to cancer progression. When the intracellular copper is overloaded, copper ions directly bind to the DLAT, promoting its lipoylation, aggregation and loss of Fe-S cluster proteins, ultimately resulting in cuproptosis. However, the association between cuproptosis related genes and prognosis in HCC have yet been illustrated. In our study, we first explored the genetic alteration of CRGs by mutation and CNV analysis in HCC cohort. CDKN2A, as one of CRGs, was found with highest mutation frequency and highly correlated with HCC prognosis. Previous study reported that approximately 8% of HCC patients harbored CDKN2A deletions, which was related to advanced stage of HCC (Khemlina et al., 2017). In addition, MTF1, another CRG with high mutation frequency, was reported to prompt HCC cell proliferation and associated with poor prognosis (Yang et al., 2022).

We then explored the differentially expressed CRGs between HCC and normal liver samples, and revealed that 9 CRGs (except FDX1) were upregulated in HCC compared to normal liver. Subsequently, we identified five significant prognostic genes by univariate cox regression. These genes have been previously reported to play pivotal roles in cancer development. CDKN2A, encoding the cyclin inhibitor p16 protein, is a new marker of poor prognosis in patients with HCC (Zhao et al., 2016), which is consistent with our study. LIPT1 encodes fatty acyltransferase 1, which regulated lipoic acid (LA) transport. LA is an important component of TCA cycle and mediated mitochondrial metabolism in cancer cells (Bingham et al., 2014). DLAT, LIPT1, and LIAS served as diagnostic biomarker in pancreatic adenocarcinoma (Wang et al., 2022). Another study revealed that zinc contributes to ovarian tumor metastasis by promoting epithelial to mesenchymal (EMT) transition through a MTF1 dependent pathway (Zhang et al., 2020). Therefore, knockout of MTF1 inhibited EMT in ovarian cancer cells (Ji et al., 2018). We also performed functional enrichment analysis by GO and KEGG. The results indicated that these genes were enriched in the TCA cycle, pyruvate metabolism, glycolysis, and HIF-1 signaling pathway, which were found to be involved in the progression of HCC. YAP-induced aerobic glycolysis, involved in HIF-1 signaling pathway, could promote the tumorigenesis of HCC (Chen et al., 2018). HIF-1 signaling pathway-related hypoxia and hyperglycemia were associated with stemness and EMT in hepatocarcinogenesis (Cui et al., 2017; Zhang et al., 2018). TCA cycle and its enzyme components mediated basal cell metabolism, thus affecting proliferation and invasion in HCC. Disrupting pyruvate uptake, which affected TCA cycle, might impair hepatocellular tumorigenesis (Tompkins et al., 2019).

In recent years, studies have been reported the relationship between programmed cell death-related phenotypes and HCC, and established corresponding prognostic models (Li and Zeng, 2022; Lu et al., 2022). However, the relationship between a newly found programmed cell death form cuproptosis and HCC was unclear. In this study, we newly developed a prognostic model based on five prognostic CRGs (namely, CDKN2A, DLAT, GLS, LIPT1, and MTF1) by performing LASSO-Cox regression, univariate and multivariate cox regression analysis. These genes were reported to correlate with HCC prognosis (Bingham et al., 2014; Wang et al., 2022). Both in the training and validation cohorts, this model stratified patients into low and high CRGs-score subtype and had a good performance in predicting the prognosis of HCC. Furthermore, we demonstrated that CRGs-score, as an independent prognostic signature, was associated with other clinical factors such as clinical stage and grade. Patients with higher CRGs-score showed higher clinical stage, grade, and worse outcomes. This model could also well predict 1-year and 3-year survival as shown by nomogram.

Accumulating studies suggested that copper was essential to maintain a regular immune response and affected tumor immunity (Kakuda et al., 2020; Zheng et al., 2020). Cuproptosis promoted tumor invasion, progression, and metastasis by impairing the anti-tumor immune responses regulated by immune cells. In our study, the immune infiltration analysis revealed that immune cells such as CD8^+^ T-cell, gamma delta T-cell, nature killer cells, macrophage, mast cells were decreased in the high CRGs-score group. Furthermore, immunity pathway analysis showed that inflammation promoting pathway was significantly enriched in low CRGs-score. Other studies revealed that inflammatory response induced copper uptake and imbalance copper concentration that promoted tumorigenesis (Liao et al., 2020; Jiang et al., 2021). Previous studies reported that lower cytolytic activity score had worse clinical outcome (Wakiyama et al., 2018; Gao et al., 2020). Here, we found that low CRGs-score had a higher score in cytolytic activity than high CRGs-score group, indicating potential better outcome. In addition, the upregulation of both type Ⅰ and type Ⅱ interferons (IFN) response, which was involved in activating the antiviral innate immune response, was observed in low CRGs-score group. Consistently, previous studies showed that upregulation of type Ⅰ IFN promoted recruitment of NK cell, and that upregulation of type Ⅱ IFN response promoted polarization of macrophages to M1 phenotype (Lee and Ashkar, 2018; Wang et al., 2018). Additionally, we analyzed the immune checkpoint genes expression between the high and low CRGs-score groups. Notably, the expression level of PD-1 and PD-L1 were significantly enhanced in high CRGs-score group. Recent studies have found that intra-tumoral copper ion regulated PD-L1 expression, resulting in tumor-specific T-cell exhaustion and immune evasion (Voli et al., 2020; Chen et al., 2021). Additionally, Cu (Ⅱ) upregulated PD-L1 expression by enhancing GSK3β phosphorylation and inhibiting PARP1 activity and ultimately inhibited T-cell infiltration. The combination of DSF/Cu(Ⅱ) and anti-PD-1 antibody showed an additive effect that slowed tumor growth in mice. (Zhou et al., 2019). These results suggested that HCC patients with low CRGs-score were probably associated with anti-tumor immunity.

Some studies have reported that the copper-related cell metabolism was a potential therapeutic target for tumors (Oliveri, 2022). In this study, we predicted the response of 10 drugs in HCC. We found that high CRGs-score subtype patients were more sensitive to 5-Fluorouracil (thymidylate synthase inhibitor), Sunitinib (receptor tyrosine kinase inhibitor), Gemcitabine, and Bleomycin than low CRGs-score subtype patients.

In summary, this study systematically revealed the landscape of molecular alterations and immune infiltration of cuproptosis-related genes in HCC. We developed a CRGs-score prognostic model including these five prognostic biomarkers (CDKN2A, DLAT, GLS, LIPT1, and MTF1), which showed a good effectiveness in predicting the survival outcome in HCC cohort. Furthermore, the higher CRGs-score was correlated with advanced stage, higher grade, worse survival, and immune deficiency. We also validated these results by cell line model and mouse model. Finally, we analyzed the sensitivity of 10 drugs for pharmacological therapeutics related to cuproptosis. Our scoring model may contribute to personalized therapy for HCC patients.

## Data Availability

The original contributions presented in the study are included in the article/Supplementary Material, further inquiries can be directed to the corresponding author.
