# Supplementary material for: A novel cuproptosis-related prognostic gene signature and validation of differential expression in hepatocellular carcinoma
Source: Front Pharmacol. 2023 Jan 10;13:1081952. doi: 10.3389/fphar.2022.1081952 (PMC9871247; doi:10.3389/fphar.2022.1081952)
Supplement: Supplementary file 2 [file DataSheet5.docx]

Supplementary Table 5 | The primers used in real-time PCR assays.

| Number | Name | Primer (5 to 3) |
| --- | --- | --- |
| 1 | DLAT-HUMAN-F | CAGCTACTCCTGCTGGACCAAA |
| 2 | DLAT-HUMAN-R | GGTGATTCTACCATCTGGTCCTG |
| 3 | dlat-MOUSE-F | CTGAGTGAAGGAGACTTGCTGG |
| 4 | dlat-MOUSE-R | TCCCAGAGGAACATCCCTTGTG |
| 5 | GLS-HUMAN-F | CAGAAGGCACAGACATGGTTGG |
| 6 | GLS-HUMAN-R | GGCAGAAACCACCATTAGCCAG |
| 7 | gls-MOUSE-F | CAGAAGGCACAGACATGGTTGG |
| 8 | gls-MOUSE-R | CAAGGTGGCAGCCATCACACTT |
| 9 | CDKN2A-HUMAN-F | CTCGTGCTGATGCTACTGAGGA |
| 10 | CDKN2A-HUMAN-R | GGTCGGCGCAGTTGGGCTCC |
| 11 | cdkn2a-MOUSE-F | TGTTGAGGCTAGAGAGGATCTTG |
| 12 | cdkn2a-MOUSE-R | CGAATCTGCACCGTAGTTGAGC |
| 13 | LIPT1-HUMAN-F | CAGGAACAGCTTCTAAGATCGGC |
| 14 | LIPT1-HUMAN-R | AGCAGTGGCATTGCTCCTGATC |
| 15 | lipt1-MOUSE-F | CTTCTTCGCTGAAGAGCCCGTA |
| 16 | lipt1-MOUSE-R | CTTCACAGGTCAGCGTGGAATC |
| 17 | MTF1-HUMAN-F | GAGGCTTCACACAGGGAAAACG |
| 18 | MTF1-HUMAN-R | GCTTTTCCACAGCCATCGTGATC |
| 19 | mtf1-MOUSE-F | GGCTGTGAGAAGGCGTTCAACA |
| 20 | mtf1-MOUSE-R | ATGTGCTTCCGCAGGTCACTGA |
